# Robotic Non-Destructive Testing

**DOI:** 10.3390/s22197654

**Published:** 2022-10-09

**Authors:** Carmelo Mineo, Yashar Javadi

**Affiliations:** 1 Institute for High-Performance Computing and Networking, National Research Council, 153, 90146 Palermo, Italy; 2 Department of Electronic & Electrical Engineering, University of Strathclyde, Glasgow G1 1XW, UK; 3 Department of Design, Manufacturing & Engineering Management, University of Strathclyde, Glasgow G1 1XW, UK

## 1. Introduction

Non-destructive testing (NDT) and evaluation (NDE) are commonly referred to as the vast group of analysis techniques used in civil, medical, and industrial sectors to evaluate the properties of materials, tissues, components, or structures without causing any damage. NDT/NDE are vital to ensure the integrity of critical parts and social safety. Automation offers many benefits for NDT to cope with increasing demands, including improved reliability and higher inspection speeds. Additionally, robots enable inspection positions that are not easily accessible to human operators to be reached and enable humans to be removed from potentially dangerous environments. However, the perceived complexity and high costs have limited the adoption of automation for NDT. As a result, the full potential that could be derived from the seamless integration of robotic platforms with sensors, actuators, and software has not been fully explored; it could still revolutionise the way automated NDT is performed and conceived. Robots are often operated by predefined tool paths generated through offline path-planning software applications. The recent advancements in electronics, robotics, sensor technology and software pave the way for new developments in automated NDT and data-driven autonomous robotic inspections in several sectors. This Special Issue aimed to attract the latest research outcomes in the field of robotic NDT. It comprises eleven high-quality papers. Five papers relate to inspection systems based on robotic fixed-base manipulators. Three research articles are associated with in-process inspection in manufacturing applications (robotic wire-arc welding and additive manufacturing). Four papers report research advancements in mobile robotic-enabled sensing. The remaining two papers focus on novel developments in data visualisation and analysis.

## 2. Overview of Contribution

Among the five papers related to inspection systems based on robotic fixed-base manipulators, three are associated with in-process inspection in robotic wire-arc welding and additive manufacturing. Zhang et al. [1] introduce a novel seam tracking technique for robotic welding. The method is proven effective, providing a reference for future seam tracking research. Vasilev et al. [2] present the development and deployment of an advanced multi-robot system for automated welding and in-process NDE. Complete external positional control is achieved in real time, allowing on-the-fly motion correction based on multi-sensory input. This approach can enable in-process weld repair, leading to higher production efficiency, reduced rework rates, and lower production costs. Zimermann et al. [3] introduce a synchronised multi-robot Wire + Arc Additive Manufacturing and NDE cell aiming to achieve in-process defect detection, enable possible in-process repair, and prevent the costly scrappage or reworking of completed defective builds. A novel high-temperature-capable, dry-coupled phased array ultrasound transducer roller-probe device is used for the NDE inspection. The dry-coupled sensor is tailored for coupling with an as-built high-temperature surface at an applied force and speed.

The other two papers on fixed-base robotic arms introduce a novel algorithm for complete-surface-finding [4] and an automated system for the real-time eddy current inspection of nuclear assets [5]. The work presented in the first article enables the robot-assisted ultrasonic testing of unknown surfaces within a single pass, a significant advancement toward fully autonomous inspection systems. The latter paper introduces a system capable of delivering an eddy current array to detect stress corrosion cracking on a nuclear canister. The variation in the lift-off of the eddy current array is innovatively minimised using a force–torque sensor, a padded flexible probe, and a feedback control system.

Four papers focus on mobile robotic applications. Zhang et al. [6] introduce a flexible design and defect detection method for a multi-sensor, wall-climbing robot used to inspect petrochemical tanks. The results show that the robot can move safely and stably on a vertical tank surface and complete precise automatic detection. Rubiales et al. [7] present a crawling mechanism using a soft-tentacle gripper integrated into an unmanned aerial vehicle for pipe inspection in industrial environments. The objective was to allow the aerial robot to perch and crawl along a pipe, minimising energy consumption and performing contact inspection. This paper introduces the design of the soft limbs of the gripper and the internal mechanism that allows movement along pipes. The other two papers in this group relate to underwater robotic inspection applications. Wang et al. [8] present a novel method for use in deep-sea plankton community detection in marine ecosystems using an underwater robotic platform. This paper demonstrates that moving plankton can be accurately detected and isolated from complex dynamic backgrounds in deep-sea environments. Cetin et al. [9] present an experimental robotic setup with a Stewart platform and a robot manipulator to emulate an underwater vehicle–manipulator system. The hardware-based emulator setup consists of a robotic manipulator mounted on a parallel manipulator, known as a Stewart Platform, and a force–torque sensor attached to the end-effector of the robotic arm interacting with a pipe. Such a complete setup is useful to use when carrying out fast and numerous experiments, circumventing the difficulties in performing similar experiments and data collection with actual underwater vehicles in water tanks.

Robotic inspection systems can acquire substantial data volumes. As a result, the research into robotic NDT/NDE must also embrace some efforts to introduce new data visualisation and analysis approaches. That is the case with the two remaining papers of this Special Issue. Mineo et al. [10] introduce an image alignment method to facilitate the visualisation and analysis of robotic thermographic inspections of parts with complex geometries. This work bridges a technology gap, making thermographic inspections more deployable in industrial environments. The proposed image alignment approach can find applicability beyond thermographic non-destructive testing. Finally, Avdelidis et al. [11] propose a two-step process for the automation of defect recognition and classification from visual images. This can be used with unmanned aerial vehicles carrying an image sensor to automate the procedure and eliminate human error.

## 3. Conclusions

Robotic NDT is a fast-evolving field which exploits the constant advancements in electronics, robotics, sensor technology, software, and network interfaces. This Special Issue is a collection of eleven high-quality publications that provide a picture of some of the most commonly investigated topics in robotic-enabled sensing. In-process inspection in robotic manufacturing applications, real-time and data-driven robotic sensing, and mobile terrestrial, underwater, and aerial robotic inspection platforms are well represented. The authors have also proposed innovative solutions to improve the visualisation and analysis of large robotically collected datasets. The advancements in robotic NDT help us face new societal challenges, which the industrial sectors have encapsulated under so-called Industry 4.0. Robotic NDT must develop with the development of new tools, including autonomous robotics, virtual-twin simulations, the Internet of Things, cybersecurity, cloud computing, augmented reality, and big data. For this reason, this Special Issue is not the conclusion of a path but the prelude to upcoming collections of research outcomes.

## Data Availability

Not applicable.
